# Epigenetics in Friedreich's Ataxia: Challenges and Opportunities for Therapy

**DOI:** 10.1155/2013/852080

**Published:** 2013-02-19

**Authors:** Chiranjeevi Sandi, Sahar Al-Mahdawi, Mark A. Pook

**Affiliations:** Division of Biosciences, School of Health Sciences and Social Care, Brunel University London, Uxbridge UB8 3PH, UK

## Abstract

Friedreich's ataxia (FRDA) is an autosomal recessive neurodegenerative disorder caused by homozygous expansion of a GAA*·*TTC trinucleotide repeat within the first intron of the *FXN* gene, leading to reduced *FXN* transcription and decreased levels of frataxin protein. Recent advances in FRDA research have revealed the presence of several epigenetic modifications that are either directly or indirectly involved in this *FXN* gene silencing. Although epigenetic marks may be inherited from one generation to the next, modifications of DNA and histones can be reversed, indicating that they are suitable targets for epigenetic-based therapy. Unlike other trinucleotide repeat disorders, such as Huntington disease, the large expansions of GAA*·*TTC repeats in FRDA do not produce a change in the frataxin amino acid sequence, but they produce reduced levels of normal frataxin. Therefore, transcriptional reactivation of the *FXN* gene provides a good therapeutic option. The present paper will initially focus on the epigenetic changes seen in FRDA patients and their role in the silencing of *FXN* gene and will be concluded by considering the potential epigenetic therapies.

## 1. Introduction

FRDA is a rare autosomal recessive neurodegenerative disorder that affects approximately 1-2 in 50,000 Caucasians [1]. In 96% of FRDA patients, the disease is caused by homozygous expansion of GAA·TTC repeats in intron 1 of the *FXN* gene [2]. Unaffected individuals have up to 40 GAA·TTC repeats, with a premutation range from 41 to 65 GAA repeats. The affected individuals contain 66 to 1700 GAA·TTC repeats [3], most commonly between 600 and 900 GAA·TTC repeats. In most cases, the GAA·TTC repeat number of the smaller allele is directly related to the age of onset and the severity of the disease. However, a small proportion of patients (approximately 4%) are compound heterozygous, having one allele with a GAA·TTC repeat expansion and the other allele with an inactivating (or loss-of-function) intragenic mutation, such as a point mutation [4, 5] or a deletion/duplication [6–9]. To date, no confirmed FRDA patients have been identified without at least one GAA·TTC repeat expansion.

The exact mechanism underlying the GAA·TTC repeat expansion in FRDA is not fully understood, but evidence has been put forward for the involvement of abnormal DNA replication, transcription, or repair [10–12]. In FRDA patients, the expanded GAA·TTC repeats produce a marked reduction in the mitochondrial protein frataxin, ranging from 4% to 29% of normal levels [13]. However, asymptomatic carriers produce about 50% of frataxin protein compared to the unaffected individuals [14]. Therefore, drugs that are able to increase frataxin expression, at least to the levels of the healthy carriers, would be beneficial. Reduced levels of frataxin protein in FRDA patients are associated with an imbalance of iron-sulfur (Fe-S) cluster biosynthesis [15], mitochondrial iron accumulation in heart, spinal cord, and dentate nucleus [16–18], and increased susceptibility to oxidative stress [19]. The outcome is progressive spinocerebellar neurodegeneration, causing symptoms of incoordination, muscle weakness, and sensory loss. There is also a pathological involvement of nonneuronal tissues with cardiomyopathy as a common secondary effect and diabetes found in approximately 10% of FRDA patients [20]. At present, there is no effective therapy for FRDA, and affected individuals generally die in early adulthood from the associated heart disease. Therefore, there is a high unmet clinical need to develop a therapy for this devastating disorder.

In view of the current knowledge regarding the FRDA pathology, some effort has been put into investigating the therapeutic interventions aimed at ameliorating secondary disease effects, such as oxidative stress and mitochondrial iron accumulation. Thus far, FRDA preclinical and clinical trials using antioxidants and iron chelators have demonstrated some limited success [20]. However, a more effective therapy may be achieved by targeting the immediate effects of the GAA·TTC repeat expansion mutation itself or the mechanisms by which the GAA·TTC repeat expansion induces the impairment of frataxin expression. Although these mechanisms are currently not known, two nonexclusive hypotheses have been put forward. Firstly, it has been suggested that the GAA·TTC repeat expansion may adopt abnormal non-B DNA structures (triplexes or “sticky DNA”) or DNA·RNA hybrid structures (R loops), which impede the process of RNA polymerase and thus reduce *FXN* gene transcription [21, 22]. Secondly, there is an evidence originally from position effect variegation (PEV) studies in transgenic mice that GAA·TTC repeat expansions can produce heterochromatin-mediated gene silencing effects [23]. Consistent with the latter hypothesis, several FRDA disease-related epigenetic changes have been identified in the immediate vicinity of the expanded GAA·TTC repeats of the *FXN* gene, and these changes will be discussed further in this paper.

## 2. Epigenetic Changes in FRDA

Epigenetic mechanisms, which include DNA methylation, histone modification, chromatin remodeling, and noncoding RNAs, result in heritable changes in gene expression that do not involve changes in DNA sequence. Epigenetic mechanisms play a crucial role in silencing or activation of many genes during development. Recognition of the role of epigenetics in human disease started with oncology, but it has now extended to other disciplines, such as neurodevelopment and neurodegenerative disorders, including Alzheimer disease (AD), Parkinson disease (PD), Huntington disease (HD), fragile X syndrome (FRAXA), and FRDA. Although epigenetic-based silencing of genes is a complicated process, three main steps have been described [24]. Firstly, the specific silencing complexes move towards the DNA sequence that is to be inactivated. Secondly, the inhibition of RNA polymerases or other nuclear enzymes takes place. Thirdly, perhaps the most crucial step in epigenetic silencing is the propagation of silent chromatin into the daughter cells [24]. It is not certain how the chromatin modifications, or “epigenetic marks,” that are established during transcription can be inherited by daughter cells. Although most of the histone modifications are reversible by epigenetic manipulation (epigenetic therapy), an effective target-specific method of reversing these modifications at a particular gene locus is yet to be achieved. The potential role of epigenetic mechanisms in FRDA was initially highlighted by the finding that long GAA·TTC repeats were able to suppress the expression of a nearby heterochromatin-sensitive cell surface reporter gene (hCD2) in transgenic mice by a phenomenon called position effect variegation (PEV) [23]. Further studies have subsequently led to the identification of epigenetic changes, including DNA methylation and histone deacetylation and methylation modifications, which may be involved in *FXN* gene silencing in FRDA.

### 2.1. DNA Methylation

DNA methylation is a covalent modification of DNA by the addition of methyl residues to cytosine bases in DNA. It is the most widely studied epigenetic mechanism in several diseases, especially cancer, providing a stable gene silencing mechanism that plays an essential role in regulating gene expression and chromatin architecture. The process of DNA methylation is carried out by DNA methyltransferase (DNMT) enzymes, which catalyze the covalent addition of a methyl group from *S*-adenosylmethionine (SAM) to the 5′ position of cytosine, predominantly within CpG dinucleotides ([25], reviewed in [26]). In mammals, the DNMT family includes three functional proteins: DNMT1, DNMT3a, and DNMT3b [27], with the most abundant being DNMT1 [25]. DNMT1 preferentially methylates hemi-methylated DNA and is thus responsible for methylation during DNA replication [28]. It plays a key role in imprinting and X-chromosome inactivation during embryogenesis [29]. DNMT3a and DNMT3b have an equal preference for hemimethylated and nonmethylated DNA and so have been classified as *de novo* methyltransferases [30]. They are responsible for *de novo* DNA methylation during embryogenesis [30, 31].

Studies that have investigated the DNA methylation profiles of transcriptionally silenced genes have revealed a strong correlation between promoter DNA methylation and transcriptional silencing. However, it has also been reported that intragenic DNA methylation can contribute to transcriptional gene silencing [32]. In addition, genome-wide studies in cancer cells have highlighted the fact that the genes that are already silenced by Polycomb complexes are more susceptible to DNA methylation compared to other genes ([33, 34], reviewed in [35]). This indicates that gene silencing by chromatin conformational changes may precede DNA methylation. Similar studies are now being performed at the *FXN* locus to unravel the role of DNA methylation in FRDA (Table 1).

Initial investigations of DNA methylation within the *FXN* gene have revealed the hypermethylation of specific CpG sites upstream of the GAA·TTC repeat sequence in FRDA patient-derived lymphoblastoid cells compared to cells derived from unaffected individuals [36]. However, such Epstein-Barr virus-transfected lymphoblastoid cells are known to frequently develop different DNA methylation patterns compared to those of primary peripheral blood leukocytes [37]. Furthermore, FRDA is a systemic disorder that is known to have differentially affected tissues and cell types. To address these issues, Al-Mahdawi et al. (2008) studied FRDA patient autopsy brain, heart, and cerebellum, the most clinically relevant tissues in FRDA. This study revealed significantly increased DNA methylation at the upstream region of the GAA·TTC repeats [38], consistent with previously published data [36]. Similar DNA methylation changes were also identified in brain, heart, and cerebellum tissues of two lines of FRDA YAC transgenic mice (YG8 and YG22) [38]. However, the degree of DNA methylation in the transgenic mice was not as severe as seen in FRDA patients, possibly because the GAA·TTC repeats in the transgenic mice (<250 GAA·TTC repeats) are smaller than those in FRDA patients (>700 GAA·TTC repeats).

**Figure 1:**
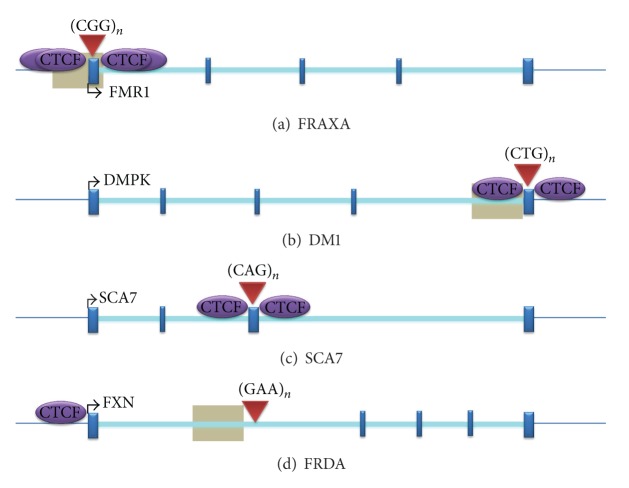
The position of DNA methylation and CTCF binding sites within TNR expansion loci. (a) FRAXA, (b) DM1, (c) SCA7, and (d) FRDA. Grey boxes represent regions of disease-associated DNA methylation. Arrow marks represent the direction of transcription. Red triangles indicate the position of repeats. This image was adapted from [139].

The level of DNA methylation was also evaluated in a large cohort of FRDA patients by the bisulfite-based EpiTYPER MassARRAY technique [39]. This study showed that the level of DNA methylation in FRDA patients is significantly elevated, especially the upstream of the GAA·TTC repeats compared to normal individuals [39], in line with the previously published data [36, 38]. It was also reported that there is no difference in DNA methylation between male and female cohorts of FRDA patients, indicating no gender specificity of DNA methylation for FRDA. Furthermore, another study has shown that the degree of DNA methylation in FRDA patients positively correlates with the length of the GAA·TTC repeats and inversely correlates with the age of the disease onset [40]. Thus, FRDA can now be grouped together with other trinucleotide repeat (TNR) expansion diseases in which an association with DNA methylation has also been reported, including FRAXA [41], myotonic dystrophy type I (DM1) [42], spinocerebellar ataxia type 1 (SCA1) [43], and spinocerebellar ataxia type 7 (SCA7) [44] (Figure 1).

### 2.2. Histone Modifications

The nucleosome, a basic subunit of chromatin structure in eukaryotes, consists of an octamer of two copies of each of the four histone proteins, H2A, H2B, H3, and H4, along with a 147 bp of DNA [45]. Histone proteins contain a globular C-terminal domain and an unstructured N-terminal tail. The remarkable feature of the histone tail is that it can contain many different modified residues. Histone modifications can regulate gene expression in several ways, by making the genetic loci more or less accessible to the transcriptional machinery. 

Although DNA methylation is considered to be a stable epigenetic modification, acetylation/deacetylation and methylation/demethylation of histone proteins play more flexible roles in the transcriptional regulation. Since histone acetylation was first reported in 1964 [46], more than 60 different types of histone modifications have been found [47]. Among these modifications, acetylation and methylation of histones at lysine (and arginine) residues are highly dynamic and are involved in several neurological disorders. The histone acetylation at lysine residues is regulated by two distinct families of enzymes with opposing action, histone acetyltransferases (HATs) and histone deacetylases (HDACs). Similarly, histone lysine methylation is controlled by histone methyltransferases (HMTs) and histone demethylases (e.g., LSD1 and JmjC), which have been linked to a number of cellular processes including DNA repair, replication, and transcriptional activation and repression [45]. Transcriptional repression of genes occurs by the deacetylation and the methylation of histone tails followed by the methylation of CpG dinucleotides by one of three DNA methyltransferases (DNMT1, DNMT3a, and DNMT3b), resulting in DNA with high levels of CpG methylation [48]. 

Based on the homologies to yeast HDACs, 18 different HDAC enzymes have been identified in mammals, and these have been divided into four classes [49–53] (Table 2). Class I consists of HDACs 1, 2, 3, and 8, which are similar to yeast RPD3 deacetylase. Class II is further divided into class IIa, which consists of HDACs 4, 5, 7, and 9, and class IIb, which consists of HDACs 6 and 10. Class II HDACs have homology to the yeast HDAC *Had-1* gene. Class III HDACs include sirtuins 1–7, also known as sirtuins, which have homology to the yeast *Sir2* gene. Lastly, class IV HDACs, which consist of only one HDAC, HDAC 11, do not have a significant homology with either class I or class II HDACs.

In recent years, advanced high-throughput techniques have improved our ability to understand the role of the epigenetic mechanisms in the pathogenesis of several neurological disorders. Epigenetic changes, which may be due to abnormally functioning HDACs, are already implicated in several neurological disorders, such as DM1 [54], FRAXA [55, 56], and spinal muscular atrophy (SMA) [57], which result in the dysregulation of the acetylation state of the chromatin. Initial findings suggested that FRDA is caused by expanded GAA·TTC repeats, which trigger an abnormal DNA structure [11, 58, 59]. However, recent studies have indicated that FRDA may also be caused by increased levels of DNA methylation, decreased histone acetylation, and increased histone methylation [36, 38, 60–62]. 

**Figure 2:**
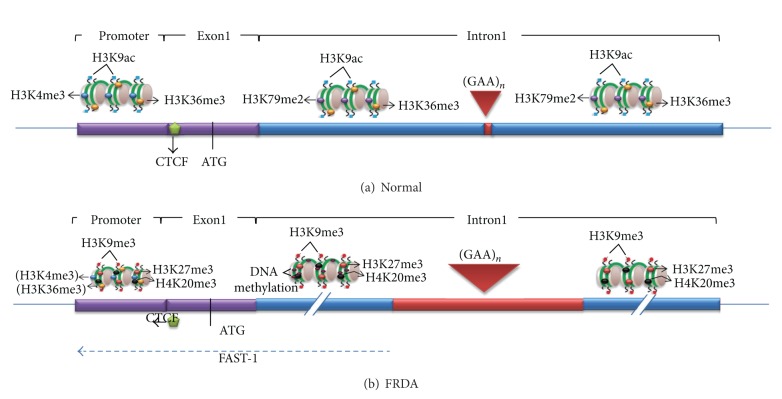
The *FXN* chromatin organization in normal individuals and FRDA patients. (a) In normal individuals, the promoter contains H3K4me3 and H3K36me3, while downstream regions contain H3K79me2 and H3K36me3, marks for transcription initiation and elongation, respectively. All regions contain H3K9ac, a mark for active open chromatin. There is CTCF binding at the 5′-UTR. (b) In FRDA, depletion of CTCF may trigger the *FAST-1* antisense transcription that may lead to the deacetylation of histones and the increase of H3K9me3 at the promoter and other regions of the gene. However, the levels of H3K4me3 and H3K36me3 are not substantially changed at the promoter (indicated by brackets), suggesting that there may be little deficiency of transcription initiation. The repressive histone marks, H3K27me3, H4K20me3 and H3K9me3, are observed throughout the gene, but most prominently at the upstream GAA repeat region, along with an increased DNA methylation. There are also reduced levels of H3K36me3 and H3K79me2 at the upstream GAA region, indicative of a defect of transcription elongation in FRDA.

Histone modifications at the *FXN* locus were first identified by the Gottesfeld lab, which reported lower levels of several acetylated H3 and H4 lysine residues, together with increased di- and trimethylation of H3K9 in the upstream GAA·TTC regions of FRDA lymphoblastoid cells [60]. Since then, other epigenetic modifications have been reported in FRDA using multiple cell types and animal models (Table 1). Greene et al. reported increased H3K9me2 levels within *FXN* intron 1 in FRDA lymphoblastoid cells [36]. Al-Mahdawi et al. reported histone modification changes at the promoter, downstream, and upstream of the GAA·TTC repeats in FRDA patient brain tissues and in an FRDA YAC transgenic mouse model [38]. This study revealed that several histone protein residues were hypoacetylated in the vicinity of the *FXN* gene, especially H3K9, in both FRDA patients and FRDA YAC transgenic mice. There is also a consistently increased H3K9 di- and trimethylation of FRDA brain tissue in all the three regions of the *FXN* gene. Furthermore, De Biase et al. reported that FRDA patient fibroblasts have significantly higher levels of H3K27me3 and H3K9me3 at the *FXN* 5′-UTR region, coupled with elevated levels of heterochromatin protein 1 (HP1), compared to those of normal fibroblasts [63]. In mammals, it is generally accepted that heterochromatin is associated with the hypoacetylation of certain histone proteins, mainly H3K9, and increased histone methylation levels, primarily H3K9me3, H3K27me3, and H4K20me3 [64–67]. In FRDA cells, increases in all of these histone modifications have been identified within the *FXN* gene, predominantly at the region immediately upstream of the expanded GAA·TTC repeats, indicating that the *FXN* gene is under a form of heterochromatin silencing machinery (Figure 2) [38, 60, 61, 63, 68, 69]. On the other hand, histone modifications such as H3K4me3, H3K36me3, and H3K79me3 are associated with a more open chromatin state and active gene expression. H3K4me3 is particularly associated with the initiation of gene transcription, while H3K36me3 and H3K79me3 are associated with the elongation of gene transcription. Recent studies have reported decreased levels of H3K36me3 and H3K79me3 at the upstream and the downstream GAA repeat regions of the *FXN* gene in FRDA cells, indicating that there is a defect in the transcription elongation [61, 68, 69]. Decreased levels of H3K4me3 have also been identified at the upstream GAA repeat region, but not at the promoter region, which suggests a more pronounced defect of the postinitiation and elongation stages of *FXN* gene expression rather than an early transcription initiation defect (Figure 2) [61, 68, 69]. In summary, there is a good evidence that the reduction of frataxin protein expression in FRDA is primarily caused by GAA repeat expansion-induced transcriptional blockage. However, the exact mechanism remains elusive and the potential defects in frataxin translation have not yet been discounted [70, 71].

### 2.3. The Role of Antisense Transcription and CTCF

Recent completion of the ENCODE project has shown that the human genome is comprised of approximately 21,000 protein coding genes, occupying only 3% of the genome [72, 73]. However, a total of 76% of the genome is transcribed, and most of this transcriptional output is made up of noncoding RNAs (ncRNAs) [72, 74–76]. Mammalian ncRNAs have been increasingly recognised as important regulators that are associated with various processes including RNA interference, imprinting, alternative splicing, and transcriptional inhibition [77–81]. 

Antisense transcription is a phenomenon where the opposite strand (antisense strand) to the protein coding strand (sense strand) is transcribed. Antisense RNA has been ascribed to roles in several molecular mechanisms, including the regulation of gene expression. Recent studies have shown that antisense transcripts can be detected in various genes, including the nonpathogenic alleles, known as natural antisense transcripts (NATs). Antisense transcripts can also be associated with microsatellite repeat expansion diseases such as HD [82], FRAXA [83, 84], SCA7 [85], SCA8 [86], and DM1 [54, 87]. In general, the level of antisense transcription is significantly lower than that of the coding sense transcripts. Nevertheless, multiple reports have recently shown that antisense transcripts can be involved in either the inhibition of the same gene from where they originate (*cis*-acting) or the inhibition of genes at different locations *(trans*-acting) [88–90]. Therefore, the study of antisense transcription in gene silencing machinery may provide further insight into the mechanisms of neurodegenerative disorders, including FRDA. To identify the presence of any antisense transcript in FRDA, De Biase and colleagues [63] performed a strand-specific reverse transcription PCR using a primer located upstream of *FXN* transcription start site 3 (TSS3). This study revealed the significantly increased levels of frataxin antisense transcript 1 (*FAST1*) at the vicinity of the transcription start site (TSS) of the *FXN* gene [63]. Furthermore, elevated levels of HP1 were identified at this locus in FRDA patient-derived fibroblasts compared to the fibroblasts of unaffected individuals [63]. It has been suggested that these aberrant features in FRDA may be associated with the depletion of CCCTC-binding factor (CTCF) that eventually leads to the disease-associated epigenetic changes for the transcriptional repression of the *FXN* gene. However, it is still unclear how CTCF is depleted in FRDA and how this leads to *FXN* gene silencing. 

CTCF is a highly conserved 11-zinc finger (ZF) nuclear protein, originally recognised as a transcription factor that binds to avian and mammalian *MYC* promoters [91]. CTCF is involved in a variety of transcriptional regulatory functions, including transcriptional activation, transcriptional repression, and genomic imprinting [92]. Additionally, CTCF is reported to have a role in inducing RNA polymerase II mediated alternative splicing [93]. DNA methylation typically prevents the binding of CTCF. However, recent studies have revealed that CTCF can also prevent the spreading of DNA methylation and thus maintains DNA methylation-free zones in the genome [94, 95]. Furthermore, CTCF binding sites have been identified in the repeat expansion flanking regions of several TNR disorders, such as FRAXA [84], DM1 [96], and SCA7 [44] (Figure 1). For FRDA, CTCF binding sites have been identified in the 5′-UTR region of the *FXN* gene in fibroblasts [63] and in cerebellum tissues (R. Mouro Pinto, S. Al-Mahdawi, and M. Pook unpublished observations) (Figure 1). The loss of CTCF binding at the DM1 CTG expansion is associated with the spread of heterochromatin and local CpG methylation [54]. Similarly, the GAA·TTC repeat expansion in FRDA is also associated with the depletion of CTCF binding in the 5′-UTR region of the *FXN* gene, which may trigger the enrichment of heterochromatin formation and DNA methylation in the upstream GAA region, although the 5′-UTR region of the *FXN* gene does not show any apparent increase in DNA methylation [36, 38, 63]. These findings support the hypothesis that CTCF prevents the spread of heterochromatin at the TNR loci, although further analysis is still required to evaluate the series of events occurring in the context of chromatin conformational changes, CTCF binding modulation, and DNA methylation for each specific TNR expansion disease.

## 3. Epigenetic Therapy for FRDA

The finding that many human diseases, including cancer, have an epigenetic aetiology has encouraged the development of a new treatment option that can be termed “epigenetic therapy.” Advances in epigenetic research have identified several drug compounds that specifically target enzymes involved in the alteration of the epigenetic states of genes, thus leading to the activation of the previously silenced genes ([60, 61], reviewed in [97]). Such drug compounds have discovered that the alteration of DNA methylation patterns and the modification of histones and several of these agents are currently being tested in clinical trials. Epigenetic therapies have already been used widely for cancer treatment, and their use is now extending to other diseases. FRDA is a disorder that currently has no effective therapy. However, since FRDA is associated with several epigenetic changes that result in a partial deficiency of frataxin mRNA and protein, it is suggested that reversing the epigenetic changes to upregulate frataxin expression may prove to be an effective therapy. Indeed, such epigenetic therapy would have two distinct advantages in FRDA: (a) a negative immune response to increased frataxin protein would be unlikely, since the body is already exposed to residual frataxin levels and (b) only a slight increase in frataxin levels may be needed to have a significant clinical effect, since heterozygous FRDA carriers are phenotypically normal.

### 3.1. DNA Demethylating Agents

Since FRDA and other neurodegenerative disorders are associated with the increased levels of DNA methylation, one can propose the use of DNA demethylating agents to reduce DNA methylation and thereby activate the previously silenced genes. DNA demethylating agents inhibit the methylation of DNA by the nonspecific inhibition of DNMTs. DNA demethylating agents are generally divided into two categories, nucleoside analogue DNMT inhibitors and nonnucleoside analogue DNMT inhibitors. Nucleoside analogue DNMT inhibitors, which include 5-azacytidine (5-aza-CR or Vidaza), 5-aza-2′-deoxycytidine (5-aza-CdR or Decitabine), and Zebularine, are analogues of cytosine, the nucleoside base that is methylated by DNMTs. 5-aza-CdR, which is an FDA-approved drug, has been tested in several phases I, II, and III clinical trials finding the most promising benefits in leukaemia patients [98], especially those affected by myelodysplastic syndrome (MDS) [99–101]. The treatment of lymphoblastoid cells from FRAXA patients with 5-aza-CdR, either alone [102] or in combination with HDAC inhibitors [103] efficiently reverses the FMR1 promoter hypermethylation and restores mRNA and protein levels to normal. This has led to the consideration of DNA demethylating agents as a potential therapy for neurodegenerative disorders. Thus far, there have been no reports of describing the use of DNA demethylating agents as a therapeutic approach for FRDA. However, our lab has recently studied the effect of several DNA demethylating agents on FRDA human and transgenic mouse primary fibroblasts (C. Sandi, unpublished observations). Our preliminary findings show 1- to 2-fold increases of *FXN* expression in FRDA transgenic mouse cells but decreased *FXN* expression in human FRDA cells, following the treatment with DNA demethylating agents (5-aza-CdR and Zebularine). Therefore, our preliminary results suggest that DNA demethylating agents are not likely to be a useful therapy for FRDA. The discrepancy between our findings in mouse and human cells is possibly due to a difference in the regulatory mechanisms of gene expression between the two species, indicating a need for further investigation. Finally, sequence-specific DNA demethylating agents, such as the oligonucleotide antisense inhibitor MG98 [104–107], may be useful for future therapeutic approaches in reducing the specific DNA methylation. In addition, the uses of naturally available green tea and derivatives have also shown beneficial effects in reducing the DNA methylation in various cancers [108–111]. Therefore, consideration of these compounds, especially those that are already in clinical trials, may be promising for future FRDA therapy.

### 3.2. Histone Deacetylase (HDAC) Inhibitors

Over the past decade, substantial progress has been made in the development of drugs that target the epigenetic changes in chromatin. Several HDAC inhibitors have been developed, ranging from the complicated chemical structures of bacterial or fungal origin, such as trichostatin A (TSA), to simple compounds such as butyrate. HDAC inhibitors can affect transcription by inducing the acetylation of histones, transcriptional factors, and other proteins that regulate transcription. They act primarily by increasing the global histone acetylation, followed by transcriptional activation of the epigenetically silenced genes through the relaxation of chromatin conformation, although some HDAC inhibitors may also promote the acetylation of nonhistone proteins [112]. 

In view of the recent identification of alterations in histone acetylation in FRDA, it has been postulated that the reversal or the inhibition of these histone modifications could represent a potential therapeutic route for FRDA [60, 113]. An initial study to screen for frataxin-increasing compounds first demonstrated a small effect of the general HDAC inhibitor sodium butyrate on *FXN* gene activity using an EGFP reporter cell line [114]. Subsequently, the treatment of FRDA lymphoblastoid cells using a selection of commercially available HDAC inhibitors revealed that only the benzamide compound BML-210 produced a significant increase of *FXN* mRNA expression [60], although other HDAC inhibitors showed a more pronounced increase of histone acetylation without any increase in *FXN* expression, indicating a degree of compound specificity for *FXN* gene silencing. Further studies using multiple cell types and mice identified three pimelic o-aminobenzamide compounds, *106*, *136*, and *109*, each of which has undergone investigations to determine safety, efficacy, and pharmacokinetic profile in short-term treatments of FRDA patient-derived cells and mice [62, 115–118] and a long-term treatment in FRDA YAC transgenic mice [71]. Compound *109*, which emerged as the most promising compound for FRDA treatment, is currently undergoing early clinical trials. However, other HDAC inhibitors such as nicotinamide [119], sirtinol [120], splitomicin [121], LBH589 [122], and oxamflatin [123] have shown positive effects in other diseases including cancer and/or neurodegenerative disorders, and these compounds may also be considered for future FRDA therapy. 

Since DNA methylation and histone modifications are both known to act epigenetically, it would perhaps be beneficial to use DNA demethylating agents and HDAC inhibitors together to investigate the potential for a more pronounced synergistic effect on increasing frataxin expression. A synergistic effect of DNA demethylating agents and HDAC inhibitors has previously been shown in the treatment of several cancers [124, 125] and neurodegenerative disorders [103]. Small noncoding RNAs, such as microRNAs, have also been implicated in several neurodegenerative disorders including AD [126], HD [127], and FRDA [128]. The elevated levels of miR-886-3p in FRDA are believed to be associated with the downregulation of the *FXN* gene, and the use of anti-miR-886-3p or the HDAC inhibitor 4b alone has been shown to partially reverse *FXN* gene repression by reducing miR-886-3p levels [128]. Therefore, it would be interesting to investigate the combined effect of anti-microRNA and HDAC inhibitor compounds on the activation of *FXN* gene transcription. 

### 3.3. Antigene RNA- (agRNA-) Based Therapies

RNA interference (RNAi) is a posttranscriptional phenomenon, where sequence-specific gene silencing is achieved by chromatin remodelling which can be triggered by a small pool of double-stranded RNAs with approximately 21 to 28 nucleotides in length [129]. Antigene RNAs (agRNAs) are small duplex RNAs with 19 bp length (2-nt overhang at 3′ end) that target gene promoters, and depending on the target sequence and the cell type, agRNAs can either silence [130–132] or activate the gene transcription [133–135]. Since agRNAs target in a sequence specific manner, it may be possible to modulate agRNAs to activate the gene expression in disease-associated genes where gene activation is essential, as with FRDA. Importantly, agRNAs can target either sense or antisense strands and coding or noncoding RNA transcripts [134]. Therefore, considering the use of agRNA to activate the *FXN* gene by targeting the *FXN* promoter or the *FAST1* transcript may be useful, since both mechanisms may be involved in reversing *FXN* gene silencing and thus FRDA pathology. 

## 4. Conclusion and Future Studies

Due to the identification of several epigenetic changes, FRDA can now be considered as an epigenetic disease. It remains to be seen whether further epigenetic marks, such as the recently identified 5-hydroxymethyl cytosine residue, may also eventually prove to have an effect on the regulation of *FXN* gene expression [136, 137]. The epigenetic marks already implicated in FRDA disease pathology have enabled the development of drugs that can target these changes and partially reverse the disease pathology. However, epigenetic therapies are generally nonspecific, with off-target effects, and the lack of specific drugs that target the *FXN* gene locus to reverse the epigenetic changes may require further consideration. Since FRDA is a multisystem disorder, it may be useful to simultaneously administer two or more drugs to examine the synergistic treatment effects. To identify novel epigenetic-based FRDA therapeutic compounds for future testing, various drug screening systems have been developed and are being utilised in several labs, including our own lab (see review [138]). Furthermore, to develop an effective system for testing potential FRDA therapies, cell and animal models are considered essential, and several model systems are currently under investigation (see review [138]).

## Figures and Tables

**Table 1:** The summary of DNA methylation, histone methylation, and histone acetylation changes in multiple cell types and animal models of FRDA.

Chromatin change	Location	Patients/cell type/animal model	Reference(s)
DNA methylation ↑	GAA upstream	Lymphoblasts, FRDA YAC transgenic mice, and primary lymphocytes	[36, 38–40]

H3K4me2/3 ↓	*FXN* promoter and exon 1	Lymphoblasts	[61, 68, 69]
	GAA upstream	Lymphoblasts	[68, 69]
	GAA downstream	Lymphoblasts	[68, 69]

H3K9me2/3 ↑	*FXN* 5′-UTR/promoter	Primary fibroblasts and lymphoblasts	[63, 68]
	GAA upstream	Lymphoblasts, FRDA YAC transgenic mice, and KIKI mice	[38, 60–62, 68, 69]
	GAA downstream	FRDA patients, FRDA YAC transgenic mice, and lymphoblasts	[38, 60, 61, 68, 69]

H3K27me3 ↑	*FXN* 5′-UTR/promoter	Primary fibroblasts and lymphoblasts	[63, 68]
	GAA upstream	Lymphoblasts	[68]
	GAA downstream	Lymphoblasts	[68]

H3K36me3 ↓	GAA upstream	Lymphoblasts	[61, 68, 69]
	GAA downstream	Lymphoblasts	[61, 68, 69]

H3K79me2 ↓	GAA upstream	Lymphoblasts	[68]
	GAA downstream	Lymphoblasts	[68]

H4K20me3 ↑	GAA upstream	Lymphoblasts	[68]
	GAA downstream	Lymphoblasts	[68]

H4K5ac ↓	*FXN* promoter	Lymphoblasts	[69]
	GAA upstream	Lymphoblasts and KIKI mice	[60, 62, 69]
	GAA downstream	FRDA patients, FRDA YAC transgenic mice, and lymphocytes	[38, 62, 69]

H3K9ac ↓	*FXN* promoter	FRDA patient brain tissue and lymphoblasts	[38, 69]
	GAA upstream	FRDA patients, FRDA YAC transgenic mice, lymphoblasts cells, and KIKI mice	[38, 60, 69]
	GAA downstream	FRDA patients, FRDA YAC transgenic mice, and lymphoblasts	[38, 60, 69]

H4K8ac ↓	GAA upstream	Lymphoblasts, FRDA patients, and KIKI mice	[38, 60, 62, 69]
	GAA downstream	Lymphoblasts and FRDA patients	[60, 69]

H4K12ac ↓	*FXN* promoter	Lymphoblasts	[60]
	GAA upstream	Lymphoblasts, FRDA patients, and FRDA YAC transgenic mice	[38, 60]
	GAA downstream	Lymphoblasts, FRDA patients, and FRDA YAC transgenic mice	[38, 60]

H3K14ac ↓	*FXN* promoter	FRDA patients	[38]
	GAA upstream	KIKI mice and lymphoblasts	[62, 69]
	GAA downstream	FRDA YAC transgenic mice and lymphoblasts	[69]

H4K16ac ↓	*FXN* promoter	Lymphoblasts	[62, 69]
	GAA upstream	Lymphoblasts, FRDA patients, FRDA YAC transgenic mice, and KIKI mice	[38, 60, 62, 69]
	GAA downstream	Lymphoblasts, FRDA patients, FRDA YAC transgenic mice, and KIKI mice	[38, 60, 62, 69]

↓: reduced, ↑: increased, H: histone, K: lysine, me2: dimethylation, me3: trimethylation, ac: acetylation, and HP: heterochromatin protein.

**Table 2:** The classification of HDACs in mammals.

Class	HDACs	Localisation
Class I	HDAC1	Nucleus
	HDAC2	Nucleus
	HDAC3	Nucleus
	HDAC8	Nucleus

Class IIa	HDAC4	Nucleus/cytoplasm
	HDAC5	Nucleus/cytoplasm
	HDAC7	Nucleus/cytoplasm
	HDAC9	Nucleus/cytoplasm

Class IIb	HDAC6	Cytoplasm
	HDAC10	Nucleus/cytoplasm

Class III	SIRT1-7	Nucleus/cytoplasm

Class IV	HDAC11	Nucleus/cytoplasm

## References

[1] Cossée M, Schmitt M, Campuzano V (1997). Evolution of the Friedreich’s ataxia trinucleotide repeat expansion: founder effect and premutations. *Proceedings of the National Academy of Sciences of the United States of America*.

[2] Campuzano V, Montermini L, Moltò MD (1996). Friedreich’s ataxia: autosomal recessive disease caused by an intronic GAA triplet repeat expansion. *Science*.

[3] Pandolfo M (2002). The molecular basis of Friedreich ataxia. *Advances in Experimental Medicine and Biology*.

[4] Cossee M, Durr A, Schmitt M (1999). Friedreich's ataxia: point mutations and clinical presentation of compound heterozygotes. *Annals of Neurology*.

[5] Gellera C, Castellotti B, Mariotti C (2007). Frataxin gene point mutations in Italian Friedreich ataxia patients. *Neurogenetics*.

[6] Deutsch EC, Santani AB, Perlman SL (2010). A rapid, noninvasive immunoassay for frataxin: utility in assessment of Friedreich ataxia. *Molecular Genetics and Metabolism*.

[7] Evans-Galea MV, Corben LA, Hasell J (2011). A novel deletion-insertion mutation identified in exon 3 of *FXN* in two siblings with a severe Friedreich ataxia phenotype. *Neurogenetics*.

[8] Zühlke CH, Dalski A, Habeck M (2004). Extension of the mutation spectrum in Friedreich’s ataxia: detection of an exon deletion and novel missense mutations. *European Journal of Human Genetics*.

[9] Anheim M, Mariani LL, Calvas P (2012). Exonic deletions of *FXN* and early-onset Friedreich ataxia. *Archives of Neurology*.

[10] Chandok GS, Patel MP, Mirkin SM, Krasilnikova MM (2012). Effects of Friedreich's ataxia GAA repeats on DNA replication in mammalian cells. *Nucleic Acids Research*.

[11] Sakamoto N, Ohshima K, Montermini L, Pandolfo M, Wells RD (2001). Sticky DNA, a self-associated complex formed at long GAA*·*TTC repeats in intron 1 of the frataxin gene, inhibits transcription. *Journal of Biological Chemistry*.

[12] Ezzatizadeh V, Pinto RM, Sandi C (2012). The mismatch repair system protects against intergenerational GAA repeat instability in a Friedreich ataxia mouse model. *Neurobiology of Disease*.

[13] Campuzano V, Montermini L, Lutz Y (1997). Frataxin is reduced in Friedreich ataxia patients and is associated with mitochondrial membranes. *Human Molecular Genetics*.

[14] Pianese L, Turano M, Lo Casale MS (2004). Real time PCR quantification of frataxin mRNA in the peripheral blood leucocytes of Friedreich ataxia patients and carriers. *Journal of Neurology, Neurosurgery and Psychiatry*.

[15] Bradley JL, Blake JC, Chamberlain S, Thomas PK, Cooper JM, Schapira AHV (2000). Clinical, biochemical and molecular genetic correlations in Friedreich’s ataxia. *Human Molecular Genetics*.

[16] Waldvogel D, van Gelderen P, Hallett M (1999). Increased iron in the dentate nucleus of patients with Friedrich's ataxia. *Annals of Neurology*.

[17] Koeppen AH, Michael SC, Knutson MD (2007). The dentate nucleus in Friedreich’s ataxia: the role of iron-responsive proteins. *Acta Neuropathologica*.

[18] Foury F, Cazzalini O (1997). Deletion of the yeast homologue of the human gene associated with Friedreich’s ataxia elicits iron accumulation in mitochondria. *FEBS Letters*.

[19] Wong A, Yang J, Cavadini P (1999). The Friedreich’s ataxia mutation confers cellular sensitivity to oxidant stress which is rescued by chelators of iron and calcium and inhibitors of apoptosis. *Human Molecular Genetics*.

[20] Schulz JB, Boesch S, Bürk K (2009). Diagnosis and treatment of Friedreich ataxia: a European perspective. *Nature Reviews Neurology*.

[21] Grabczyk E, Mancuso M, Sammarco MC (2007). A persistent RNA*·*DNA hybrid formed by transcription of the Friedreich ataxia triplet repeat in live bacteria, and by T7 RNAP in vitro. *Nucleic Acids Research*.

[22] Wells RD (2008). DNA triplexes and Friedreich ataxia. *FASEB Journal*.

[23] Savellev A, Everett C, Sharpe T, Webster Z, Festenstein R (2003). DNA triplet repeats mediate heterochromatin-protein-1-sensitive variegated gene silencing. *Nature*.

[24] Beisel C, Paro R (2011). Silencing chromatin: comparing modes and mechanisms. *Nature Reviews Genetics*.

[25] Robertson KD (2001). DNA methylation, methyltransferases, and cancer. *Oncogene*.

[26] Goffin J, Eisenhauer E (2002). DNA methyltransferase inhibitors—state of the art. *Annals of Oncology*.

[27] Bestor TH (2000). The DNA methyltransferases of mammals. *Human Molecular Genetics*.

[28] Pradhan S, Bacolla A, Wells RD, Roberts RJ (1999). Recombinant human DNA (cytosine-5) methyltransferase. I. Expression, purification, and comparison of novo and maintenance methylation. *Journal of Biological Chemistry*.

[29] Beard C, Li E, Jaenisch R (1995). Loss of methylation activates Xist in somatic but not in embryonic cells. *Genes and Development*.

[30] Okano M, Bell DW, Haber DA, Li E (1999). DNA methyltransferases Dnmt3a and Dnmt3b are essential for de novo methylation and mammalian development. *Cell*.

[31] Watanabe D, Suetake I, Tada T, Tajima S (2002). Stage- and cell-specific expression of Dnmt3a and Dnmt3b during embryogenesis. *Mechanisms of Development*.

[32] Lorincz MC, Dickerson DR, Schmitt M, Groudine M (2004). Intragenic DNA methylation alters chromatin structure and elongation efficiency in mammalian cells. *Nature Structural and Molecular Biology*.

[33] Gal-Yam EN, Egger G, Iniguez L (2008). Frequent switching of polycomb repressive marks and DNA hypermethylation in the PC3 prostate cancer cell line. *Proceedings of the National Academy of Sciences of the United States of America*.

[34] Schlesinger Y, Straussman R, Keshet I (2007). Polycomb-mediated methylation on Lys27 of histone H3 pre-marks genes for de novo methylation in cancer. *Nature Genetics*.

[35] Jones PA (2012). Functions of DNA methylation: islands, start sites, gene bodies and beyond. *Nature Reviews Genetics*.

[36] Greene E, Mahishi L, Entezam A, Kumari D, Usdin K (2007). Repeat-induced epigenetic changes in intron 1 of the frataxin gene and its consequences in Friedreich ataxia. *Nucleic Acids Research*.

[37] Sugawara H, Iwamoto K, Bundo M (2011). Comprehensive DNA methylation analysis of human peripheral blood leukocytes and lymphoblastoid cell lines. *Epigenetics*.

[38] Al-Mahdawi S, Pinto RM, Ismail O (2008). The Friedreich ataxia GAA repeat expansion mutation induces comparable epigenetic changes in human and transgenic mouse brain and heart tissues. *Human Molecular Genetics*.

[39] Evans-Galea MV, Carrodus N, Rowley SM (2012). *FXN* methylation predicts expression and clinical outcome in Friedreich ataxia. *Annals of Neurology*.

[40] Castaldo I, Pinelli M, Monticelli A (2008). DNA methylation in intron 1 of the frataxin gene is related to GAA repeat length and age of onset in Friedreich ataxia patients. *Journal of Medical Genetics*.

[41] Naumann A, Hochstein N, Weber S, Fanning E, Doerfler W (2009). A distinct DNA-methylation boundary in the 5′- upstream sequence of the FMR1 promoter binds nuclear proteins and is lost in fragile X syndrome. *American Journal of Human Genetics*.

[42] López Castel A, Nakamori M, Tomé S (2011). Expanded CTG repeat demarcates a boundary for abnormal CpG methylation in myotonic dystrophy patient tissues. *Human Molecular Genetics*.

[43] Dion V, Lin Y, Hubert L, Waterland RA, Wilson JH (2008). Dnmt1 deficiency promotes CAG repeat expansion in the mouse germline. *Human Molecular Genetics*.

[44] Libby RT, Hagerman KA, Pineda VV (2008). CTCF cis-regulates trinucleotide repeat instability in an epigenetic manner: a novel basis for mutational hot spot determination. *PLoS Genetics*.

[45] Kouzarides T (2007). Chromatin modifications and their function. *Cell*.

[46] Allfrey VG, Faulkner R, Mirsky AE (1964). Acetylation and methylation of histones and their possible role in the regulation of RNA synthesis. *Proceedings of the National Academy of Sciences of the United States of America*.

[47] Tan M, Luo H, Lee S (2011). Identification of 67 histone marks and histone lysine crotonylation as a new type of histone modification. *Cell*.

[48] Razin A (1998). CpG methylation, chromatin structure and gene silencing—a three-way connection. *EMBO Journal*.

[49] Blander G, Guarente L (2004). The Sir2 family of protein deacetylases. *Annual Review of Biochemistry*.

[50] Bhalla KN (2005). Epigenetic and chromatin modifiers as targeted therapy of hematologic malignancies. *Journal of Clinical Oncology*.

[51] Marks PA, Dokmanovic M (2005). Histone deacetylase inhibitors: discovery and development as anticancer agents. *Expert Opinion on Investigational Drugs*.

[52] Glaser KB (2007). HDAC inhibitors: clinical update and mechanism-based potential. *Biochemical Pharmacology*.

[53] Pan LN, Lu J, Huang B (2007). HDAC inhibitors: a potential new category of anti-tumor agents. *Cellular & Molecular Immunology*.

[54] Cho DH, Thienes CP, Mahoney SE, Analau E, Filippova GN, Tapscott SJ (2005). Antisense transcription and heterochromatin at the DM1 CTG repeats are constrained by CTCF. *Molecular Cell*.

[55] Coffee B, Zhang F, Ceman S, Warren ST, Reines D (2002). Histone modifications depict an aberrantly heterochromatinized FMR1 gene in fragile X syndrome. *American Journal of Human Genetics*.

[56] Kumari D, Usdin K (2010). The distribution of repressive histone modifications on silenced FMR1 alleles provides clues to the mechanism of gene silencing in fragile X syndrome. *Human Molecular Genetics*.

[57] Kernochan LE, Russo ML, Woodling NS (2005). The role of histone acetylation in SMN gene expression. *Human Molecular Genetics*.

[58] Sakamoto N, Chastain PD, Parniewski P (1999). Sticky DNA: self-association properties of long GAA*·*TTC repeats in R*·*R*·*Y triplex structures from Friedreich’s ataxia. *Molecular Cell*.

[59] Sakamoto N, Larson JE, Iyer RR, Montermini L, Pandolfo M, Wells RD (2001). GGA*·*TCC-interrupted triplets in long GAA*·*TTC repeats inhibit the formation of triplex and sticky DNA structures, alleviate transcription inhibition, and reduce genetic instabilities. *Journal of Biological Chemistry*.

[60] Herman D, Jenssen K, Burnett R, Soragni E, Perlman SL, Gottesfeld JM (2006). Histone deacetylase inhibitors reverse gene silencing in Friedreich’s ataxia. *Nature Chemical Biology*.

[61] Punga T, Bühler M (2010). Long intronic GAA repeats causing Friedreich ataxia impede transcription elongation. *EMBO Molecular Medicine*.

[62] Rai M, Soragni E, Jenssen K (2008). HDAC inihibitors correct frataxin deficiency in a Friedreich ataxia mouse model. *PLoS One*.

[63] De Biase I, Chutake YK, Rindler PM, Bidichandani SI (2009). Epigenetic silencing in Friedreich ataxia is associated with depletion of CTCF (CCCTC-binding factor) and antisense transcription. *PLoS One*.

[64] Martin C, Zhang Y (2005). The diverse functions of histone lysine methylation. *Nature Reviews Molecular Cell Biology*.

[65] Kourmouli N, Jeppesen P, Mahadevhaiah S (2004). Heterochromatin and tri-methylated lysine 20 of histone H4 in animals. *Journal of Cell Science*.

[66] Jenuwein T (2006). The epigenetic magic of histone lysine methylation: delivered on 6 July 2005 at the 30th FEBS Congress in Budapest, Hungary. *FEBS Journal*.

[67] Sims RJ, Nishioka K, Reinberg D (2003). Histone lysine methylation: a signature for chromatin function. *Trends in Genetics*.

[68] Kim E, Napierala M, Dent SY (2011). Hyperexpansion of GAA repeats affects post-initiation steps of *FXN* transcription in Friedreich's ataxia. *Nucleic Acids Research*.

[69] Kumari D, Biacsi RE, Usdin K (2011). Repeat expansion affects both transcription initiation and elongation in Friedreich ataxia cells. *Journal of Biological Chemistry*.

[70] Acquaviva F, Castaldo I, Filla A (2008). Recombinant human erythropoietin increases frataxin protein expression without increasing mRNA expression. *Cerebellum*.

[71] Sandi C, Pinto RM, Al-Mahdawi S (2011). Prolonged treatment with pimelic o-aminobenzamide HDAC inhibitors ameliorates the disease phenotype of a Friedreich ataxia mouse model. *Neurobiology of Disease*.

[72] Bernstein BE, Birney E, Dunham I (2012). An integrated encyclopedia of DNA elements in the human genome. *Nature*.

[73] Harrow J, Frankish A, Gonzalez JM (2012). GENCODE: the reference human genome annotation for The ENCODE Project. *Genome Research*.

[74] Qi P, Du X (2013). The long non-coding RNAs, a new cancer diagnostic and therapeutic gold mine. *Modern Pathology*.

[75] Ponting CP, Belgard TG (2010). Transcribed dark matter: meaning or myth?. *Human Molecular Genetics*.

[76] Djebali S, Davis CA, Merkel A (2012). Landscape of transcription in human cells. *Nature*.

[77] Lavorgna G, Dahary D, Lehner B, Sorek R, Sanderson CM, Casari G (2004). In search of antisense. *Trends in Biochemical Sciences*.

[78] Pasquinelli AE, Ruvkun G (2002). Control of developmental timing by microRNAs and their targets. *Annual Review of Cell and Developmental Biology*.

[79] Delaval K, Feil R (2004). Epigenetic regulation of mammalian genomic imprinting. *Current Opinion in Genetics and Development*.

[80] Morris KV, Santoso S, Turner AM, Pastori C, Hawkins PG (2008). Bidirectional transcription directs both transcriptional gene activation and suppression in human cells. *PLoS Genetics*.

[81] Munroe SH, Lazar MA (1991). Inhibition of c-erbA mRNA splicing by a naturally occurring antisense RNA. *Journal of Biological Chemistry*.

[82] Chung DW, Rudnicki DD, Yu L, Margolis L (2011). A natural antisense transcript at the Huntington's disease repeat locus regulates HTT expression. *Human Molecular Genetics*.

[83] Khalil AM, Faghihi MA, Modarresi F, Brothers SP, Wahlestedt C (2008). A novel RNA transcript with antiapoptotic function is silenced in fragile X syndrome. *PLoS One*.

[84] Ladd PD, Smith LE, Rabaia NA (2007). An antisense transcript spanning the CGG repeat region of FMR1 is upregulated in premutation carriers but silenced in full mutation individuals. *Human Molecular Genetics*.

[85] Sopher BL, Ladd PD, Pineda VV (2011). CTCF regulates ataxin-7 expression through promotion of a convergently transcribed, antisense noncoding RNA. *Neuron*.

[86] Moseley ML, Zu T, Ikeda Y (2006). Bidirectional expression of CUG and CAG expansion transcripts and intranuclear polyglutamine inclusions in spinocerebellar ataxia type 8. *Nature Genetics*.

[87] Yu Z, Teng X, Bonini NM (2011). Triplet repeat-derived siRNAs enhance RNA-mediated toxicity in a drosophila model for myotonic dystrophy. *PLoS Genetics*.

[88] Chen J, Sun M, Kent WJ (2004). Over 20% of human transcripts might form sense-antisense pairs. *Nucleic Acids Research*.

[89] Wang XJ, Gaasterland T, Chua NH (2005). Genome-wide prediction and identification of cis-natural antisense transcripts in Arabidopsis thaliana. *Genome Biology*.

[90] Carmichael GG (2003). Antisense starts making more sense. *Nature Biotechnology*.

[91] Lobanenkov VV, Nicolas RH, Adler VV (1990). A novel sequence-specific DNA binding protein which interacts with three regularly spaced direct repeats of the CCCTC-motif in the 5′-flanking sequence of the chicken c-myc gene. *Oncogene*.

[92] Phillips JE, Corces VG (2009). CTCF: master weaver of the genome. *Cell*.

[93] Shukla S, Kavak E, Gregory M (2011). CTCF-promoted RNA polymerase II pausing links DNA methylation to splicing. *Nature*.

[94] Engel N, Thorvaldsen JL, Bartolomei MS (2006). CTCF binding sites promote transcription initiation and prevent DNA methylation on the maternal allele at the imprinted H19/Igf2 locus. *Human Molecular Genetics*.

[95] Filippova GN, Cheng MK, Moore JM (2005). Boundaries between chromosomal domains of X inactivation and escape bind CTCF and lack CpG methylation during early development. *Developmental Cell*.

[96] Filippova GN, Thienes CP, Penn BH (2001). CTCF-binding sites flank CTG/CAG repeats and form a methylation-sensitive insulator at the DM1 locus. *Nature Genetics*.

[97] Yoo CB, Jones PA (2006). Epigenetic therapy of cancer: past, present and future. *Nature Reviews Drug Discovery*.

[98] Jain N, Rossi A, Garcia-Manero G (2009). Epigenetic therapy of leukemia: an update. *International Journal of Biochemistry and Cell Biology*.

[99] Issa JPJ, Garcia-Manero G, Giles FJ (2004). Phase 1 study of low-dose prolonged exposure schedules of the hypomethylating agent 5-aza-2′-deoxycytidine (decitabine) in hematopoietic malignancies. *Blood*.

[100] Saba HI (2007). Decitabine in the treatment of myelodysplastic syndromes. *Therapeutics and Clinical Risk Management*.

[101] Saba HI, Wijermans PW (2005). Decitabine in myelodysplastic syndromes. *Seminars in Hematology*.

[102] Chiurazzi P, Pomponi MG, Willemsen R, Oostra BA, Neri G (1998). In vitro reactivation of the FMR1 gene involved in fragile X syndrome. *Human Molecular Genetics*.

[103] Chiurazzi P, Pomponi MG, Pietrobono R, Bakker CE, Neri G, Oostra BA (1999). Synergistic effect of histone hyperacetylation and DNA demethylation in the reactivation of the FMR1 gene. *Human Molecular Genetics*.

[104] Stewart DJ, Donehower RC, Eisenhauer EA (2003). A phase I pharmacokinetic and pharmacodynamic study of the DNA methyltransferase 1 inhibitor MG98 administered twice weekly. *Annals of Oncology*.

[105] Winquist E, Knox J, Ayoub JP (2006). Phase II trial of DNA methyltransferase 1 inhibition with the antisense oligonucleotide MG98 in patients with metastatic renal carcinoma: a National Cancer Institute of Canada Clinical Trials Group investigational new drug study. *Investigational New Drugs*.

[106] Amato RJ (2007). Inhibition of DNA methylation by antisense oligonucleotide MG98 as cancer therapy. *Clinical Genitourinary Cancer*.

[107] Plummer R, Vidal L, Griffin M (2009). Phase I study of MG98, an oligonucleotide antisense inhibitor of human DNA methyltransferase 1, given as a 7-day infusion in patients with advanced solid tumors. *Clinical Cancer Research*.

[108] Kato K, Long NK, Makita H (2008). Effects of green tea polyphenol on methylation status of RECK gene and cancer cell invasion in oral squamous cell carcinoma cells. *British Journal of Cancer*.

[109] Dou QP (2009). Molecular mechanisms of green tea polyphenols. *Nutrition and Cancer*.

[110] Yuasa Y, Nagasaki H, Akiyama Y (2009). DNA methylation status is inversely correlated with green tea intake and physical activity in gastric cancer patients. *International Journal of Cancer*.

[111] Pandey M, Shukla S, Gupta S (2010). Promoter demethylation and chromatin remodeling by green tea polyphenols leads to re-expression of GSTP1 in human prostate cancer cells. *International Journal of Cancer*.

[112] Butler R, Bates GP (2006). Histone deacetylase inhibitors as therapeutics for polyglutamine disorders. *Nature Reviews Neuroscience*.

[113] Festenstein R (2006). Breaking the silence in Friedreich’s ataxia. *Nature Chemical Biology*.

[114] Sarsero JP, Li L, Wardan H, Sitte K, Williamson R, Ioannou PA (2003). Upregulation of expression from the FRDA genomic locus for the therapy of Friedreich ataxia. *Journal of Gene Medicine*.

[115] Rai M, Soragni E, Chou CJ (2010). Two new pimelic diphenylamide HDAC inhibitors induce sustained frataxin upregulation in cells from Friedreich’s ataxia patients and in a mouse model. *PloS One*.

[116] Chou CJ, Herman D, Gottesfeld JM (2008). Pimelic diphenylamide 106 is a slow, tight-binding inhibitor of class I histone deacetylases. *Journal of Biological Chemistry*.

[117] Soragni E, Herman D, Dent SYR, Gottesfeld JM, Wells RD, Napierala M (2008). Long intronic GAA•TTC repeats induce epigenetic changes and reporter gene silencing in a molecular model of Friedreich ataxia. *Nucleic Acids Research*.

[118] Xu C, Soragni E, Chou CJ (2009). Chemical probes identify a role for histone deacetylase 3 in Friedreich’s ataxia gene silencing. *Chemistry and Biology*.

[119] Ghosh S, Feany MB (2004). Comparison of pathways controlling toxicity in the eye and brain in Drosophila models of human neurodegenerative diseases. *Human Molecular Genetics*.

[120] Ota H, Tokunaga E, Chang K (2006). Sirt1 inhibitor, Sirtinol, induces senescence-like growth arrest with attenuated Ras-MAPK signaling in human cancer cells. *Oncogene*.

[121] Biacsi R, Kumari D, Usdin K (2008). SIRT1 inhibition alleviates gene silencing in Fragile X mental retardation syndrome. *PLoS Genetics*.

[122] Garbes L, Riessland M, Hölker I (2009). LBH589 induces up to 10-fold SMN protein levels by several independent mechanisms and is effective even in cells from SMA patients non-responsive to valproate. *Human Molecular Genetics*.

[123] Kim YB, Lee KH, Sugita K, Yoshida M, Horinouchi S (1999). Oxamflatin is a novel antitumor compound that inhibits mammalian histone deacetylase. *Oncogene*.

[124] Cecconi D, Donadelli M, Dalla Pozza E (2009). Synergistic effect of trichostatin A and 5-aza-2′-deoxycytidine on growth inhibition of pancreatic endocrine tumour cell lines: a proteomic study. *Proteomics*.

[125] Luszczek W, Cheriyath V, Mekhail TM, Borden EC (2010). Combinations of DNA methyltransferase and histone deacetylase inhibitors induce DNA damage in small cell lung cancer cells: correlation of resistance with IFN-stimulated gene expression. *Molecular Cancer Therapeutics*.

[126] Cogswell JP, Ward J, Taylor IA (2008). Identification of miRNA changes in Alzheimer’s disease brain and CSF yields putative biomarkers and insights into disease pathways. *Journal of Alzheimer’s Disease*.

[127] Lee ST, Chu K, Im WS (2011). Altered microRNA regulation in Huntington's disease models. *Experimental Neurology*.

[128] Mahishi LH, Hart RP, Lynch DR, Ratan RR (2012). miR-886-3p levels are elevated in Friedreich Ataxia. *The Journal of Neuroscience*.

[129] Hannon GJ (2002). RNA interference. *Nature*.

[130] Janowski BA, Hu J, Corey DR (2006). Silencing gene expression by targeting chromosomal DNA with antigene peptide nucleic acids and duplex RNAs. *Nature Protocols*.

[131] Janowski BA, Huffman KE, Schwartz JC (2005). Inhibiting gene expression at transcription start sites in chromosomal DNA with antigene RNAs. *Nature Chemical Biology*.

[132] Morris KV, Chan SWL, Jacobsen SE, Looney DJ (2004). Small interfering RNA-induced transcriptional gene silencing in human cells. *Science*.

[133] Janowski BA, Younger ST, Hardy DB, Ram R, Huffman KE, Corey DR (2007). Activating gene expression in mammalian cells with promoter-targeted duplex RNAs. *Nature Chemical Biology*.

[134] Watts JK, Yu D, Charisse K (2010). Effect of chemical modifications on modulation of gene expression by duplex antigene RNAs that are complementary to non-coding transcripts at gene promoters. *Nucleic Acids Research*.

[135] Li LC, Okino ST, Zhao H (2006). Small dsRNAs induce transcriptional activation in human cells. *Proceedings of the National Academy of Sciences of the United States of America*.

[136] Kriaucionis S, Heintz N (2009). The nuclear DNA base 5-hydroxymethylcytosine is present in purkinje neurons and the brain. *Science*.

[137] Tahiliani M, Koh KP, Shen Y (2009). Conversion of 5-methylcytosine to 5-hydroxymethylcytosine in mammalian DNA by MLL partner TET1. *Science*.

[138] Martelli A, Napierala M, Puccio H (2012). Understanding the genetic and molecular pathogenesis of Friedreich’s ataxia through animal and cellular models. *Disease Models & Mechanisms*.

[139] Pook MA, Tatarinova T, Kerton O (2012). DNA methylation and trinucleotide repeat expansion diseases. *DNA Methylation—From Genomics to Technology*.

